# An Approach to Enhance the Conservation-Compatibility of Solar Energy Development

**DOI:** 10.1371/journal.pone.0038437

**Published:** 2012-06-07

**Authors:** D. Richard Cameron, Brian S. Cohen, Scott A. Morrison

**Affiliations:** The Nature Conservancy, San Francisco, California, United States of America; University of Oxford, United Kingdom

## Abstract

The rapid pace of climate change poses a major threat to biodiversity. Utility-scale renewable energy development (>1 MW capacity) is a key strategy to reduce greenhouse gas emissions, but development of those facilities also can have adverse effects on biodiversity. Here, we examine the synergy between renewable energy generation goals and those for biodiversity conservation in the 13 M ha Mojave Desert of the southwestern USA. We integrated spatial data on biodiversity conservation value, solar energy potential, and land surface slope angle (a key determinant of development feasibility) and found there to be sufficient area to meet renewable energy goals without developing on lands of relatively high conservation value. Indeed, we found nearly 200,000 ha of lower conservation value land below the most restrictive slope angle (<1%); that area could meet the state of California’s current 33% renewable energy goal 1.8 times over. We found over 740,000 ha below the highest slope angle (<5%) – an area that can meet California’s renewable energy goal seven times over. Our analysis also suggests that the supply of high quality habitat on private land may be insufficient to mitigate impacts from future solar projects, so enhancing public land management may need to be considered among the options to offset such impacts. Using the approach presented here, planners could reduce development impacts on areas of higher conservation value, and so reduce trade-offs between converting to a green energy economy and conserving biodiversity.

## Introduction

Climate change poses one of the greatest threats to biodiversity [1], [2]. Many species will be challenged to adapt to the magnitude and pace of the change, especially those already compromised by habitat loss and degradation [3]. Conservation of biodiversity will rely on protecting and enhancing the resilience and permeability of landscapes, to increase the viability of native species and provide them access to conditions they will need to persist in the future [4]. Efforts to reduce greenhouse gas emissions will also provide benefits to natural systems by reducing the magnitude of climate change impacts to which they need to adapt. Indeed, development of utility-scale (>1 MW) renewable energy generation facilities is a core element of a multi-faceted strategy to reduce emissions from the energy sector [5]. Yet, such facilities can have sizable footprints in terms of land area and water use [6], and so can threaten natural ecosystems directly through habitat loss and fragmentation, or indirectly through the displacement of other human land uses [7]. Therein lies a paradox of utility-scale renewable energy development: it may be necessary to reduce climate change impacts and help protect biodiversity worldwide in the future; but if not carefully planned, it could come at the expense of the viability of local species today or constrain their ability to adapt to future conditions by destroying, or creating dispersal barriers to, areas they will need in the future.

The current pace and scale of efforts to develop renewable energy sources can make it more difficult to avoid adverse ecological impacts, especially given the lack of scientific studies regarding those impacts [8]. Yet, if emissions levels are to be maintained below what some describe as “dangerous” for both natural and human systems [9], [10], conversion to renewable sources of energy needs to be rapid worldwide [11]. Interest in energy security and economic stimulus further fuels demand for renewable energy development in the United States. Utility-scale development has become a government priority at the national and subnational level, with regulatory and financial incentives to further it (examples include the National Energy Policy Act of 2005, American Reinvestment & Recovery Act of 2008) including $5.3 B in loan guarantees for three projects in California [12]. This has resulted in a boom market for renewable energy in the western United States that has overwhelmed state and federal environmental regulatory processes and permitting agencies. For example, as of November 2010, there were 22 applications to develop solar facilities on Bureau of Land Management (BLM) lands in the California deserts alone, with a cumulative footprint of nearly 78,000 ha [13].

Regulatory complexity compounds the political and market pressures. Authority for permitting new renewable energy facilities is dispersed across multiple jurisdictions depending on the technology, the size of the facility being proposed, and whether the proposed location is on public or privately-owned land. A variety of undesired consequences may result from this high political pressure and complexity, including protracted and controversial approval processes, unexpectedly high compensatory mitigation costs, and approval of projects prior to a full understanding of their cumulative environmental impact.

Decision-support tools are needed to efficiently guide projects toward areas that are commercially attractive for development, and away from areas important for biodiversity conservation and other resources. Using such tools in the early phase of project scoping would allow developers to select areas where they will be less likely to encounter environmental obstacles in the permitting process. These “low-conflict” locations could be prioritized for field investigations and possibly be eligible for expedited permitting or other incentives to promote projects on appropriate lands. Conservationists also benefit from early identification of areas with minimal conservation value as it might expedite the attainment of climate benefits and reduce the risk of their being perceived as obstructionist.

**Figure 1:**
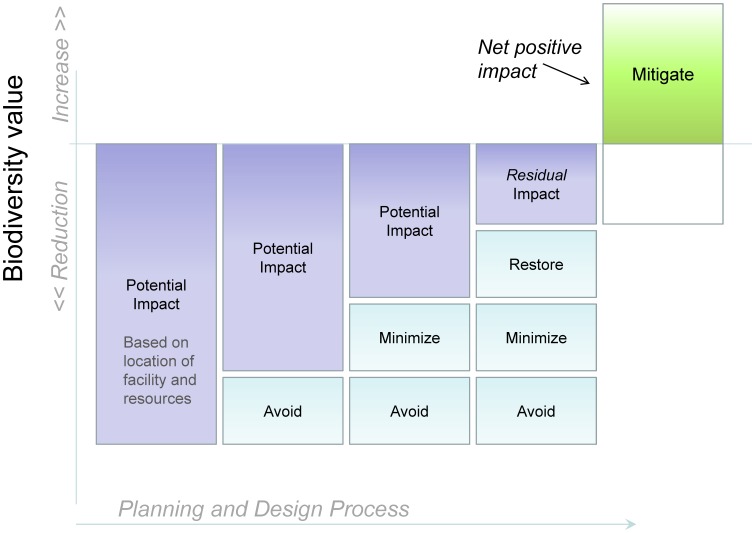
The mitigation hierarchy. Under this schema, developers advancing a project choose locations for their project that avoid environmental impacts. If impacts cannot be completely avoided, they then take steps to minimize impacts. Once impacts are minimized to the extent possible, restoration opportunities are pursued. Residual impacts not addressed by the previous steps are then offset through compensatory mitigation, using ratios that result in a net positive impact on biodiversity. Adapted from Convention on Biological Diversity 2008 [54].

Avoiding impacts through the selection of appropriate development locations and compensating for any residual impacts are core components of the “mitigation hierarchy”, a planning approach most commonly used to avoid impacts to wetlands [14], [15] (Figure 1). Adherence to this approach can help reduce adverse impacts of development, by defining resources and areas to be avoided, and outlining steps to minimize, restore, or offset unavoidable impacts. The principles of the mitigation hierarchy can be applied at a landscape scale through spatial analyses that map constraints and opportunities for both development and conservation [16]–[18]. Finding areas that are both suitable for renewable energy development and of relatively low biodiversity conservation value represents a possible “win-win” for two otherwise potentially conflicting objectives [19]. When complete avoidance of impacts is not possible, this approach can improve the conservation return of investments in compensatory mitigation, by directing it to places and efforts that also advance regional conservation goals [16], [20], [21].

Here, we illustrate how the mitigation hierarchy can be applied to characterize the degree of alignment between biodiversity conservation and electricity generation from utility-scale solar facilities. Our study focuses on the Mojave Desert, as it is the focus of intense development pressure: it offers large expanses of public lands with exceptional solar energy resources in close proximity to highly populated regions with strong markets for renewable energy. We integrate conservation values and presumed development feasibility across the desert, and illustrate how compensatory mitigation can contribute to regional conservation goals. We propose that this regional application of the mitigation hierarchy can lead to both more efficient development of renewable energy and better conservation outcomes in the Mojave Desert, and that this approach can serve as a model for resolving such conflicts more generally.

### Study Area

The Mojave Desert Ecoregion encompasses 13,013,000 ha, across four southwestern states: California (contains 56% of the ecoregion), Nevada (31%), Arizona (11%) and Utah (2%). The ecoregion is notable for its biodiversity as well as for its wilderness values and associated economic benefits [22]. There are over 400 vertebrate species that inhabit the ecoregion, with extremely high endemism especially in wetland areas, such as Ash Meadows National Wildlife Refuge, Nevada where there are 24 endemic plants and animals [23], [24]. Plant diversity in shrub communities is among the highest in North America, with potential species diversity in these communities as high as 70 species per hectare in the eastern Mojave [25]. Currently 29 species and subspecies in the Mojave Desert are listed as threatened or endangered under the federal Endangered Species Act [23]. The region has extensive public and military lands (collectively covering over 85% of the ecoregion), with 53% of the ecoregion designated for wilderness and for species habitat – such as critical habitat for the federally threatened desert tortoise (*Gopherus agassizii).*

**Figure 2:**
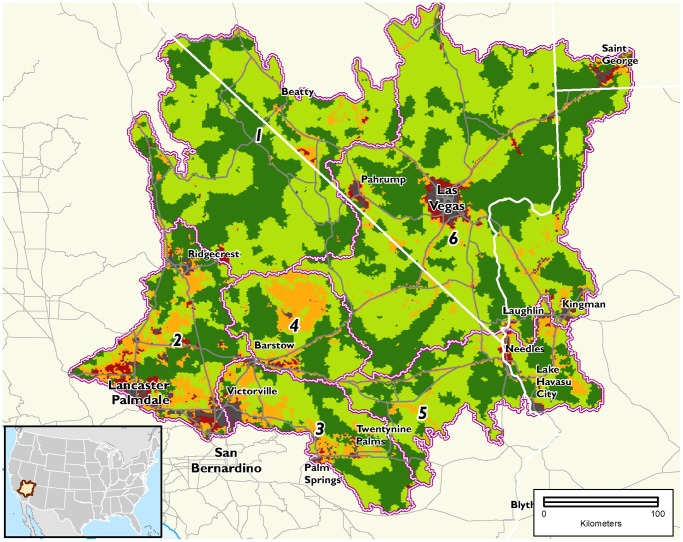
Conservation value in the Mojave Desert Ecoregion. The conservation values categories are depicted on the map as follows: dark green areas are Ecologically Core, light green are Ecologically Intact, orange are Moderately Degraded, and red are Highly Converted (adapted from Randall et al. 2010). Subregions of the Mojave Desert are shown in the purple-white outline; labels indicate the 1. Northern, 2. Western, 3. South-central, 4. Central, 5. Southeastern, and 6. Eastern subregions. Urbanized land is grey and highways are in grey lines. The location of the ecoregion in the coterminous United States is shown in the inset map.

The biodiversity input into this analysis is a characterization of *conservation value* across the Mojave Desert Ecoregion, from Randall et al.’s (2010) Mojave Ecoregional Assessment (hereafter, the Assessment) [26]. The Assessment analyzed a broad set of conservation elements, or “targets” (44 vegetation communities and 521 plant and animal taxa) and used the conservation planning software Marxan [27] to generate alternative configurations of areas to meet conservation objectives. By integrating Marxan output of priority areas, aerial photo interpretation (to assess degree of anthropogenic ground disturbance), and principles of conservation reserve design, Randall et al. classified the land into categories of high (i.e., Ecologically Core, Ecologically Intact) and low (i.e., Moderately Degraded, Highly Converted) conservation value (Figure 2). Here, we used the latter category to represent areas of lower conservation value. We note that the approach we present is flexible, and could accommodate other conservation assessments as the biodiversity input. For example, other prioritization analyses exist for individual species in the ecoregion (such as federal endangered species critical habitat units) or as habitat conservation plans for portions of the ecoregion [28]–[29]. We selected the Randall et al. 2010 conservation value assessment because it is the most recent and consistent characterization of the distribution of biodiversity and land use impacts across the whole of the ecoregion.

The Mojave Desert is also renowned for its extraordinary solar resources. An analysis of the solar energy production potential of the southwestern United States suggests that the region could supply 50% of the country’s electricity demand if fully developed [30], [31]. One of the largest collections of solar electricity facilities in the world, the Solar Energy Generating Systems (SEGS) is installed in the Mojave Desert, totaling 354 MW of installed capacity.

Certain attributes of the desert ecosystem warrant special attention in planning for industrial land uses such as energy facilities. The low productivity of the desert leads to a slow pace of soil development, plant growth, and ecological succession, and that renders it slow to recover from disturbances [32]. This limits the application of the mitigation hierarchy, in that restoration of disturbed areas is often infeasible in ecological timeframes. While restoration is a critical step for reducing impacts from infrastructure development in many ecosystems, the challenges of successful restoration in desert systems increases the importance of avoidance and minimization strategies. Mechanical disturbance of soil crusts leads to erosion and heightened susceptibility to invasion by non-native grasses and forbs [33]. Those, in turn, can result in altered fire regimes, and effectively irreversible type conversion of habitats [34]. Disturbing desert soil may also limit the degree to which it acts as a carbon sink, an ecological process that is poorly studied and the magnitude of which has only recently been characterized [35]. Solar facilities also consume water in their installation, operation, and or maintenance. Water is very limiting in the desert, with many species dependent upon either the rare surface expressions of water or the vegetation communities that draw upon subsurface flows. Although relationships between surface and ground water, as well as ground water flows and recharge rates are poorly understood, it is generally accepted that these resources are over-allocated [36]. While a full consideration of the ecological values of desert ecosystems is beyond the scope of this study (see Lovich and Ennen 2011), the integrity of soils and the scarcity of water are two key ecological attributes for planning, and potential constraints on the ability to align solar energy development and biodiversity conservation.

## Results

### Regional Opportunities to Align Energy and Conservation Goals

**Figure 3:**
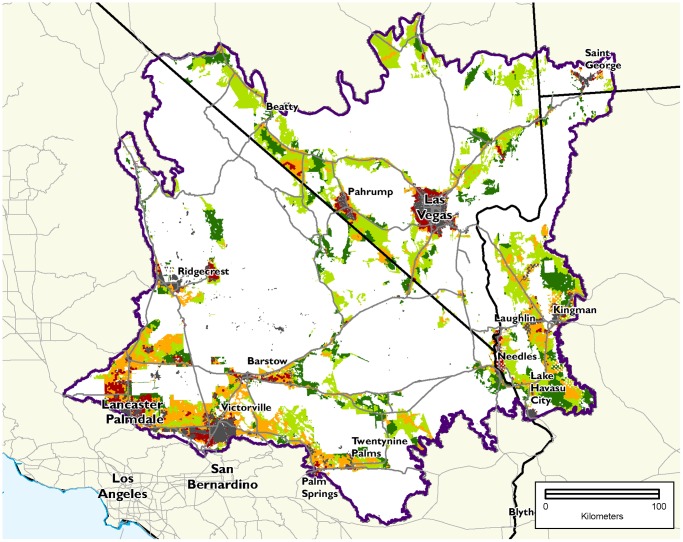
Conservation values in potentially suitable lands for solar development below 5% slope angle. Urban areas, water bodies, and lands outside of private or BLM multiple use ownerships, and areas above 5% slope were removed. Conservation value colors are the same as Figure 2. Lands in orange and red are classified as lower conservation value lands for which energy production estimates are provided in the results.

We found large areas of the Mojave Desert that are potentially suitable for the development of solar facilities that are ecologically degraded with lower regional conservation value (Figure 3). The amount of lower conservation value land that meets the development suitability criteria ranges from nearly 200,000 ha (<1% land surface slope angle) to over 740,000 ha (<5% slope) (Table 1). The level of potential compatibility between development and conservation is much greater if land with higher slope can be utilized, with nearly four times more lower conservation value land at the 5% cutoff compared to the 1%.

**Table 1:** Area (ha) of land by land ownership, conservation value, and percentage slope angle.

Owner	Ecologically Core	Ecologically Intact	Moderately Degraded	Highly Converted	Total
**Bureau of Land Management (BLM)**					
<5% slope	389,458	828,371	190,244	21,669	1,429,742
<3% slope	240,370	491,398	130,530	17,762	880,061
<1% slope	73,736	99,196	31,785	10,570	215,288
**Private Land**					
<5% slope	159,693	221,835	400,264	128,522	910,315
<3% slope	128,260	168,127	326,898	111,955	735,239
<1% slope	49,045	34,811	89,886	58,687	232,428

Areas with lower than 7 kwh/m^2^/day direct normal irradiance (DNI) were excluded from the analysis, as were legally and administratively protected areas, urban areas, and perennial water bodies. BLM land includes only undesignated land eligible for potential siting. Higher percentage slope categories are inclusive of the lower. Conservation value categories from Randall et al. 2010.

**Figure 4:**
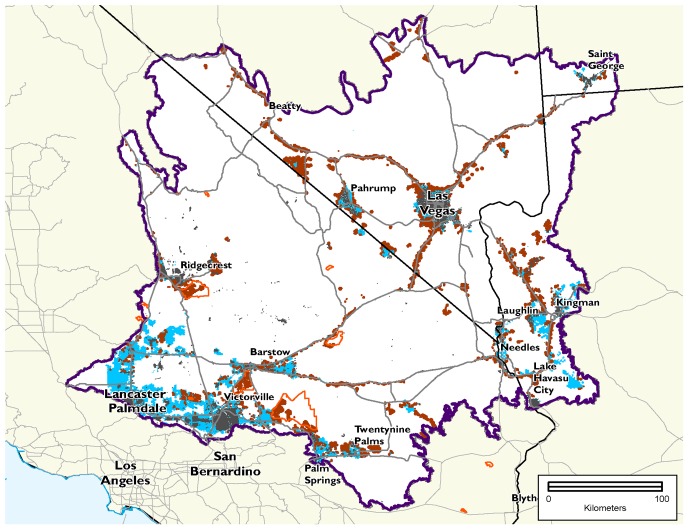
Land ownership in potentially suitable lands below 5% slope with Moderately Degraded and Highly Converted conservation value. Blue areas are private lands and dark red areas are BLM land without designation. Areas outlined in orange are designated open off-highway vehicle areas on BLM land in California, accounting for 10% of the 211,000 ha in lower conservation value on BLM land and would not be suitable for development. Conservation values adapted from Randall et al. 2010.

**Figure 5:**
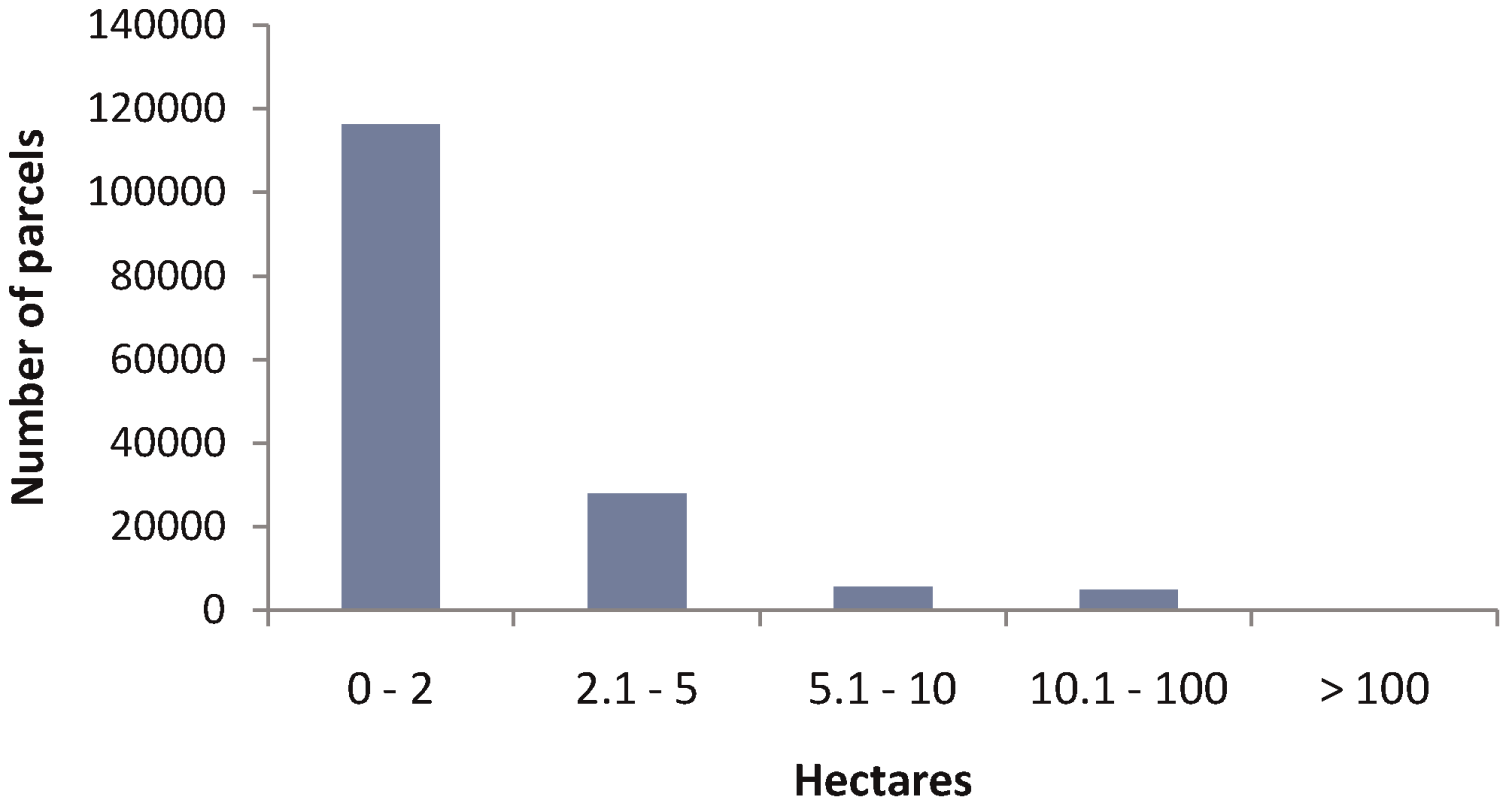
Parcel size class distribution within private lands of California that are of lower conservation value. These are only within areas that are potentially suitable for solar development below 5% slope. The presence of high rates of parcelization on private land acts as a disincentive to site large solar projects in more degraded areas.

Privately-owned parcels provide considerably more opportunity to develop on land with lower conservation value than do public lands (Figure 4, Table 1). The combined area of lower conservation value private land is 3.5 times (<1% slope) to 2.5 times (<5% slope) the area of those categories on suitable BLM land across the ecoregion. The higher degradation on private land is primarily due to agricultural land use and low density development in parts of the western Mojave in California and in the Arizona portion of the ecoregion. However, unlike BLM-managed lands, private lands are often parcelized and divided into many ownerships. In California, private lands that meet suitability criteria, are less than 5% slope and are in the lower conservation value categories, the average parcel size is 2.4 ha, with a median of 1 ha (Figure 5).

While most of the degraded areas potentially suitable for development are found on private land, BLM land also provides large areas of potential opportunity for development, with over 210,000 ha of lower conservation value land less than 5% slope across the ecoregion (Table 1, Figure 4). About 90% of those lands are available for solar use since approximately 10% (21,522 ha) are within designated off highway vehicle (OHV) open areas and thus likely to be off limits to and inappropriate for development.

### Ecoregional Impacts

**Figure 6:**
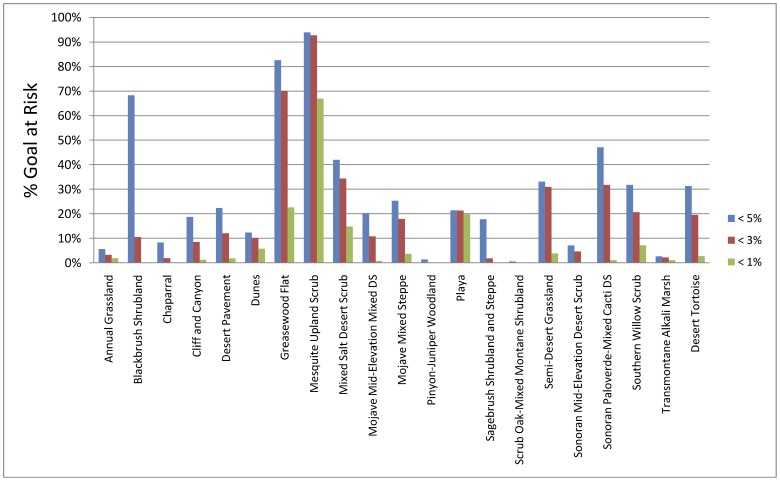
Percent of representation goals that would not be attainable if all areas potentially suitable for solar development were to be developed. The goals refer to a hypothesized amount of each habitat that needs to be managed for conservation to meet long-term viability needs for representative biodiversity of the ecoregion. Goals are based on Randall et al. 2010.

If the full extent of areas without protective designation (i.e., BLM multiple use and private lands) that are potentially suitable for solar facilities were to be opened and used for solar development, large areas of Ecologically Core and Intact (hereafter, “higher conservation value”) lands would be lost, ranging from over 250,000 ha (<1%) to 1.6 million ha (<5%) (Table 1). This extent of loss would greatly reduce the ability to meet ecoregional conservation goals (per Randall et al. 2010) for many biodiversity targets, especially if higher slopes are eligible for development (Figure 6). Some targets would face extensive loss relative to the current distribution, such as mesquite upland scrub, greasewood flats, blackbrush shrubland, and mixed salt desert scrub [37] (Figure 6). The extent of desert tortoise suitable habitat outside tortoise conservation areas in higher conservation value lands that would be lost varies considerably based on slope angle, from 90,103 ha (<1%) to over 1 M ha (<5%). The location of many of the areas at risk are in flat valleys which often connect existing conservation lands for wide-ranging species like desert bighorn sheep (*Ovis canadensis nelsoni*) [38].

In the California and Nevada portion of the Mojave, there are over 220,000 ha of solar facilities proposed as Right of Way (ROW) applications on BLM lands, including nearly 130,000 ha of Ecologically Core and Intact habitats (Table 2). The vast majority of this area – over 116,000 ha – is occupied by the ecoregion’s most widespread community, creosotebush-white bursage desert scrub (*Larrea tridentata*, *Ambrosia dumosa*). The second most extensive impact would be to Mojave mid-elevation mixed desert scrub [37] (Table 2). The desert tortoise is wide-ranging across the study area, and would directly lose 103,509 ha of Ecologically Core and Intact suitable habitat if the footprints of all current proposals on BLM lands are developed.

**Table 2:** The extent of ecological system targets that occur within BLM Right of Way applications in California or Nevada that also occur within Ecologically Core or Ecologically Intact conservation value categories (from Randall et al. 2010).

Conservation Target	Area Potentially Impacted (Ha)
Creosotebush-White Bursage Desert Scrub	116,640
Mojave Mid-Elevation Mixed Desert Scrub	7,125
Southern Willow Scrub	1,145
Mixed Salt Desert Scrub	1,082
Cliff and Canyon	1,075
Playa	699
Desert Pavement	578
Dunes	229
Greasewood Flat	165
Chaparral	110

### Supply Relative to Renewable Energy Goals

California’s 2020 Renewables Portfolio Standard (RPS) goal can be fully met without developing within the Ecologically Core or Intact lands in the ecoregion. The lower conservation value land with slopes of less than 1% (190,928 ha) could supply 107 TWh of electricity, or 180% of the renewable energy that it is estimated will be needed to meet the California RPS by the Renewable Energy Transmission Initiative (RETI) [39]. Below 3% slope, there are 587,145 ha of land, with the potential to generate 555% of the energy required, while the lower conservation value lands below 5% cutoff (740,699 ha) could supply 700% of the energy required.

### Mitigation Scenarios

**Figure 7:**
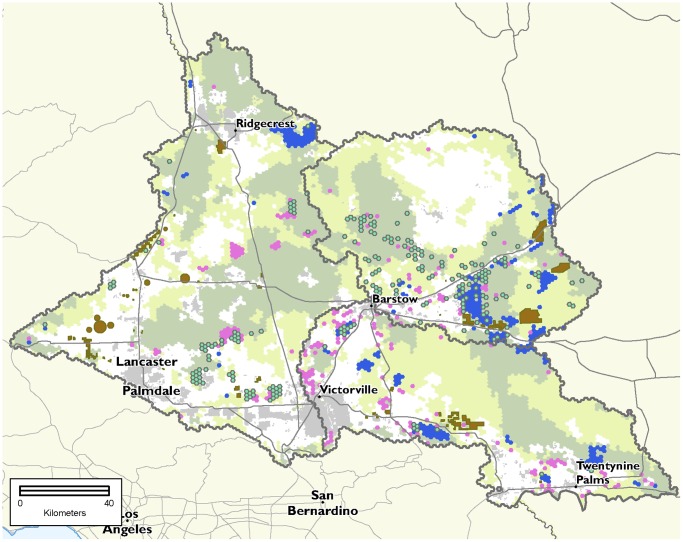
Scenarios of suitable mitigation areas using the future ratios. This map shows the private land-only (pink) and the mixed ownership (blue) scenarios, with planning units that are shared in both scenarios (teal with outline). The private land-only solution is more dispersed and was not able to offset impacts for five targets in a subregion (grey outlines, labeled in Figure 2), most notably a deficit of over 23,000 hectares of suitable desert tortoise habitat in the Central Mojave subregion, north and east of Barstow, CA. Urbanized areas are shown in light grey. The extent of Ecologically Core (darker green) and Ecologically Intact (light green) is shown for reference (adapted from Randall et al. 2010). Projects used to calculate impacts and drive mitigation demand are shown in brown.

We calculated a total footprint of 31,994 ha for proposed solar energy generation facilities under verified Right of Way applications on BLM lands and on private lands of the western, central and south-central subregions of the ecoregion. Meeting compensatory mitigation needs for these proposed projects would contribute more to regional conservation goals if mitigation is not restricted to private lands. For example, if we use the “future” mitigation ratio and restrict mitigation investment to private lands, there will not be enough higher conservation value private land in the central Mojave subregion to offset impacts for five conservation targets, including the desert tortoise, which falls short of the mitigation need by 38% (23,104 ha) (Figure 7). In contrast, if public lands are also eligible for investment, mitigation requirements under the future ratio could be met for all but two targets (playa is short by 601 ha and desert pavement is short by 30 ha) (Figure 7). Moreover, in the private land only scenario, lands selected for mitigation at both ratio levels are more fragmented than the mixed ownership scenario (as reflected in higher edge length of the full selected network, 15% higher for current ratios and 52% for future ratios). The areas selected in the private land only, current scenario are slightly more degraded (11%, as indicated by the average Marxan “cost” per selected assessment unit) than the mixed ownership solution (Table 3). This difference in degradation jumps to 60% using the future ratios, which is largely due to Marxan seeking to meet the mitigation goals for tortoise, by having to include areas that may be relatively more impacted.

The ideal arrangement of places for mitigation differs depending on what lands are available. The percentage overlap of the mitigation solutions for the mixed ownership and the private land only scenarios is low: the Jaccard similarity index [40] was 0.29 for the current mitigation ratio and 0.42 for the future ratio (Figure 7). A similar comparison of total area needed for both ownership scenarios could not be performed for the future ratio solutions because mitigation goals could not be met in the private land only, future scenario (Table 3).

**Table 3:** Performance of compensatory mitigation scenarios.

Mitigation Ratios	Eligible Land for Mitigation	Assessment Unit Cost	# of Assessment Units	Boundary Length (m)	Goals Met for All Targets?
Current	Private Core and Intact	158,999	254	1,087,545	No (1 not met)
Current	Private or BLM undesignated Core and Intact	141,084	251	717,062	No (1 not met)
Future	Private Core and Intact	447,275	457	1,862,370	No (5 not met)
Future	Private or BLM undesignated Core and Intact	324,674	531	1,617,374	No (2 not met)

Assessment unit costs are the sum of the “cost” values, a unitless index used in Marxan as a proxy for anthropogenic disturbance. The number of assessment units is the number selected in the most efficient scenario of 100 model runs. Boundary length is the total edge length of the selected assessment units and is a proxy for the dispersion of the selected network of areas. Goal attainment refers to whether the mitigation goals for the targets are met in the given scenario. See Supporting Information S2 for full description of Marxan settings.

## Discussion

We found considerable opportunity for alignment of biodiversity conservation and solar energy development objectives in the Mojave Desert. Assessed at the moderate 3% slope cutoff, over 580,000 ha of lands with lower conservation value yet presumably suitable for solar energy development currently exist across the desert, an amount that could supply over five times the energy needed to meet the projected 2020 California 33% RPS goal. Steering development to areas of lower conservation value could help reduce adverse impacts to desert ecosystems, specifically areas that are more intact and those that contain sensitive resources. Avoiding those areas will likely improve the adaptive capacity of desert species in the face of climate change and provide greater ecological resilience in the future. Prioritizing development in lower conservation value lands reduces the prospect of conflict over ecological impacts that can add cost, delay, and controversy to projects.

One striking finding from this study is the relationship between land ownership, conservation value, and “attractiveness for development.” From a conservation perspective, most of the areas that appear better suited for development are privately held, but they are often comprised of many parcels that would need to be consolidated to achieve a minimum area sufficient to support a project. From a development perspective, that parcelization creates a disincentive, especially if an alternative exists to have a more streamlined process working elsewhere with one land owner, e.g., BLM. Thus, one strategy to enhance protection of the conservation values of the Mojave Desert would be to develop policies that incentivize development on degraded private lands. We note that brownfields and areas formerly in agricultural production, but retired due to salinity, water limitations, economic considerations, or other contamination problems may present ideal locations for solar development, especially for technologies that use less groundwater than the former land use.

The approach we present can also help direct compensatory mitigation investments. By accounting for the direct impacts of a given set of proposed projects and the distribution of lands with higher conservation value, we illustrate how one can generate a portfolio of candidate areas for compensatory mitigation that meet mitigation obligations while contributing to regional conservation goals. Of course, further field assessment is required to ensure that candidate sites generated from this type of analysis are indeed suitable as mitigation. This approach can be generalized to other land uses, geographies, covered resources, and mitigation ratios and actions, and explored as a site-selection problem to optimize various social and ecological goals.

Our analysis of land ownership and conservation value also revealed a conundrum for mitigation. While the higher degradation of private lands provides opportunities to avoid or minimize adverse ecological impacts when siting projects, it also poses problems if compensatory mitigation can only be conducted on private lands. The limited supply of private lands with higher conservation values could in turn limit the amount of energy development for which impacts can be offset. We note, however, that there may be considerable opportunity to use mitigation funds to enhance the conservation management of existing public lands in the desert, through such actions as eradicating invasive species, increasing enforcement of off-highway vehicle closures, or installing tortoise exclusion fencing along roads. The desert tortoise recovery plan [41], for example, recommends numerous management actions to enhance species viability, many of which go unimplemented due to insufficient funding [42]. We emphasize that any investment of mitigation resources applied to public lands would need to result in enduring conservation outcomes and add to the current level of management activities rather than replace existing resources and agency obligations. One way to track and better ensure that investments result in enduring conservation is to change the designation of lands serving as mitigation from one that allows multiple uses to one that gives primacy to the conservation use. Ensuring additionality of mitigation-related enhanced management funding would likely involve contractual obligations and require special enforcement mechanisms within agency budgeting processes.

We underscore the importance of accounting for cumulative impacts in siting and mitigation decisions, especially in light of the increased stress that climate change will exert on desert ecosystems. The impacts of projects should not only be evaluated comprehensively regarding ecological impacts, but also examined cumulatively in the context of all of the major stressors in the desert (including but not limited to the other proposed energy projects). Because of the large area potentially impacted by long-term solar energy development (as illustrated in Figure 6), and the lack of related impact studies, a framework is needed in the near term to guide decision-making to help reduce the risk of inadvertently crossing thresholds of ecological viability [8]. The approach presented here, essentially an application of the precautionary principle, can provide that initial guidance: develop first in the least conflict areas and protect the consensus conservation areas; meanwhile, improve knowledge regarding the areas in between, so that siting and mitigation decisions in the future can be better informed as to their environmental trade-off.

Limitations of this analysis are mostly related to data quality and resolution. We underscore that this study cannot substitute for site-level assessment, or more detailed assessments of sensitive and rare species’ conservation needs (e.g., HCPs [Habitat Conservation Plans], NCCPs [the state of California’s Natural Communities Conservation Plans], endangered and threatened species recovery plans). Moreover, the map of the relative conservation value should not be construed as a development and conservation blueprint, *per se*. Randall et al. (2010) caution that because important occurrences, ecological processes or habitats of targets may occur within all of the conservation value categories, even the Highly Converted category, site-level assessment is needed to confirm suitability for development, and guide project siting, design, and mitigation. The Assessment is best used to provide general guidance to planners and industry seeking to assess the relative likelihood of environmental constraints across a broad area, in an attempt to minimize adverse permitting problems. As suitable information becomes available, the approach we present here can be implemented at a finer spatial scale for a portion of the ecoregion.

An additional limitation of our analysis is that it does not explicitly account for some key factors that influence the economic feasibility of project development. Geographic factors may affect the economic profitability of a site, such as local influences on solar radiation or the costs of ongoing maintenance to minimize damage from airborne sand. One notable factor that was beyond the scope of our study pertains to transmission. Proximity to transmission corridors that have additional capacity is an important consideration in siting new generation facilities. The relationship between transmission and generation will be important to incorporate into future refinements of this analysis utilizing the expertise of the solar industry, especially where new transmission is required to service proposed facilities. Those additional impacts should be incorporated into the overall application of the mitigation hierarchy.

In sum, we demonstrate how solar energy production goals in the Mojave Desert can be met with less adverse effect on biodiversity. The systematic approach presented here for proactively balancing solar energy production with biodiversity protection better accounts for, and so can help reduce, trade-offs. Importantly, it can also provide greater assurances to agencies, developers and conservationists that their respective goals are being met. Integrating this sort of analysis with dynamic information systems for species distributions, ecological condition and conservation investments, can help agencies and stakeholders adaptively apply the mitigation hierarchy with increasing effectiveness. This example of multi-objective planning can also be expanded and tailored to other technologies and geographies, e.g., wave energy and marine protected areas. We caution, however, that if such planning does not incorporate and accommodate all major interests and stakeholders, it may lead to displacement of one user by another, and exacerbate rather than resolve conflict. For example, our analysis did not incorporate some significant desert values, such as cultural values, recreational uses, military training, and scenic values. Accounting for this array of interests will be essential for developing the long-term conservation plan for the Mojave.

Numerous conservation and energy development planning efforts are currently underway that will affect the Mojave Desert (e.g., BLM’s Solar Energy Development Programmatic Environmental Impact Statement). The State of California is currently developing an NCCP for the state’s deserts that, like this analysis, will take into account not just those species currently listed but the full array of natural communities of the California deserts. We are hopeful that the resulting NCCP will identify areas preferred for development and conservation, and institutionalize effective regulatory mechanisms and market-based incentives to implement that plan. Ideally, those mechanisms will help ensure that siting and mitigation occur in the places most appropriate for effecting desert conservation–regardless of the underlying ownership. In the interim, we propose that a precautionary approach like that presented here could guide conservation-compatible renewable energy development in the desert.

## Materials and Methods

### Solar Energy Development Potential

We estimated solar energy potential across the Mojave Desert using the direct normal irradiance (DNI) data at 10 km resolution developed by National Renewable Energy Laboratory (NREL) and SUNY-Albany [43]. The DNI is the variable commonly used to assess the potential for concentrating solar power (CSP) installations, but is strongly correlated with solar insolation values used to plan solar photovoltaic (PV) facilities.

Development feasibility was characterized based on land ownership and management, current land use, and land surface percent slope angle, as well as solar insolation. We filtered the DNI data to include only those lands with excellent solar resource potential (annual average value of at least 7 kWh/m^2^/day ) and slope angles that bracket the maximum slope that is considered to be developable for solar energy based on current technologies (less than 1%, 3% (inclusive), and 5% (inclusive)). We calculated the slope using elevation data from the Shuttle Radar Topography Mission (SRTM) resampled from 30 meters to 90 meters resolution, and smoothed using an averaging filter by a 3 × 3 window to remove anomalies in the data [44]. To remove patches of land not large enough for utility-scale solar projects, we applied a minimum mapping unit of 100 hectares and merged all polygons below this cutoff with adjacent polygons using the ARCGIS Eliminate tool [45].

To ensure that areas already developed with residential, industrial or commercial uses were not included as potentially suitable, we created a composite “developed” land layer. For Utah, Arizona and Nevada we used data from the Southwest ReGap program [46] to represent developed land use. For California, we extracted the “urban” category from the Multi-source Land Cover data [47] to represent the footprint of areas to exclude. To minimize adjacency to urban areas, we smoothed the developed land composite using an averaging filter by a 3×3 window and removed all areas greater than 10% urbanized after smoothing. Perennial water bodies and areas that have a legal or administrative status that prevents energy development were also removed from the suitable land base. We removed the categories of land that were identified as consensus exclusion areas in California’s Renewable Energy Transmission Initiative [48] (see Supporting Information S1 for a list of these categories). We also excluded the desert tortoise conservation areas as defined by the U.S. Fish and Wildlife Service, which include areas designated as critical habitat for the desert tortoise [49]. Mohave ground squirrel (*Spermophilus mohavensis)*) conservation areas [50] were also removed because they have been proposed for exclusion by the BLM. Management status data on the location of public and private land and the relative level of conservation management were from the U.S. Geological Survey Protected Areas Data version 1.1 [51].

### Progress toward California Renewable Energy Goals

A key driver of demand for renewable energy in the Mojave Desert is the California Renewables Portfolio Standard (RPS), which mandates that investor- and publicly-owned utilities acquire 33% of their energy from renewable sources by 2020. The net amount of renewable energy that needs to come online to meet the 2020 goal will change over time and requires assumptions about the lifespan of current and future projects. We used an estimate from the California Renewable Energy Transmission Initiative [39] of 59.7 TWh which is higher than more recent estimates [52]. We calculated the potential energy generation based on the land area that is developable based on the solar insolation, slope, and land use and management filters described above, and conservation value (per Randall et al. 2010) for the whole ecoregion. We used this potential energy generation to estimate the proportion of the remaining California’s RPS goal (net short) that could be met in the Moderately Degraded and Highly Converted (hereafter, “lower conservation value”) lands in the ecoregion. We considered the California RPS as a realistic energy goal for this analysis, and we assumed that land in other states can have projects to contribute to the California RPS goal given the close proximity of many of the areas to California. To convert land area to energy output, we used the mid-point land area to energy estimate for solar thermal provided in MacDonald et al. (2009) of 3.8 ha/mw and assumed a 25% capacity factor [7].

### Development Impacts and Mitigation Opportunities

We analyzed opportunities to offset projected impacts from BLM and private land solar projects by developing mitigation scenarios that differed in 1) the type of land ownership allowed to serve as mitigation, and 2) the mitigation offset ratio. The extent of this analysis included three subregions used in the Assessment: the Western, Central, and South Central Mojave Desert (Figure 2). We used only the northern portion of the South Central subregion (dividing it based on the ecological subsection boundary [53]) because the southern portion is covered by Joshua Tree National Park and an adjacent Area of Critical Environmental Concern (ACEC), which are land designations that do not allow for development.

To estimate subregional impacts, we used the mapped or estimated footprints of proposed solar projects on private lands in Kern, San Bernardino, and Los Angeles counties within the California Mojave ecoregional boundary and the verified ROW applications for BLM lands in California [13]. For the BLM projects, we used the California verified Right of Way solar projects from a data download from November 8, 2010. For the private land projects, we used maps or available GIS data from Kern, Los Angeles and San Bernardino counties. Specifically, for Kern County projects was a spreadsheet and digital map showing the location of the facilities, acquired from the county and dated September 9, 2010. The facilities were digitized based on this map and a point GIS file was created. The area of the facility was used from the spreadsheet to buffer the point to a circle with an area the exact same size as the listed size in the table. The source for San Bernardino County projects was from April 2010 and included two pre-application projects. These were digitized based on the locations and information in a digital map acquired from the county. We mapped the projects as precisely as possible to get the approximate acreage and location based on the information available, though we were not able to map projects more accurately than the parcel boundary. For Los Angeles County, projects were mapped based on available assessor parcel numbers and parcel data acquired in December 2010 from the county. The three county data layers and the BLM ROW layer were merged into one file within the extent of the subregional area. Each project was assigned to a subregion with no projects straddling subregions. We could not identify a data source for Inyo County in the western subregion.

To estimate potential ecoregional impacts from ROW applications, we included both California and Nevada applications. We assume that the whole area within the ROW would be impacted by the proposed projects, even though in many cases the area of the ROW application exceeds the actual development footprint. We caution that these footprints represent only the direct impacts associated with the projects, not indirect effects. It is also likely that not all of these applications will be developed. However, the purpose of this portion of the study is to characterize the magnitude of the impact of solar development based on a proposed set of projects and resultant mitigation it will require in one portion of the Mojave Desert.

To derive the amount of mitigation needed for species and vegetation system targets, we calculated the extent for each vegetation type and habitat for two species of conservation interest (desert tortoise [49], Mojave ground squirrel [50]) within the ROW applications and private land projects in the subregional study area. The calculated impacts for these 45 projects were used to identify potential areas to meet compensatory mitigation needs in the most efficient configuration (based on total area, length of outer boundary of selected hexagons, and conservation suitability described below) while contributing to regional conservation goals. We used the same tool for the mitigation scenarios that was used in the Assessment, Marxan (v. 1.8.10), to identify areas that can meet mitigation needs. We ensured that potential mitigation areas would contribute to conservation goals by allowing Marxan to select only Ecologically Core or Intact areas from the Assessment, without an existing protective designation, such as Federal Wilderness areas or Areas of Critical Environmental Concern. To ensure that the mitigation areas would be ecologically similar to the impacted resources, we required the offsetting to be within the same subregion as the impact. Additional parameters and goal amounts used for Marxan scenarios are shown in Supporting Information S2.

To assess mitigation needs, we used two sets of mitigation to impact ratios. The first set was intended to mitigate for the impacts of existing proposed projects (hereafter “current”). Current ratios were based on available guidance in existing regulations and recovery plans, although we included all target ecological systems, not just those for which mitigation is required under existing laws and regulations. The second set of ratios was intended to be a proxy for potential future build out of solar projects (hereafter “future”). “Future” ratios were defined as double the “current” ratios (Table 4). This simple approach to forecasting mitigation needs can be used to design programmatic investments, such as advance mitigation. To assess the influence of land ownership on the availability of mitigation options, we ran scenarios with two alternatives: only using private land as suitable sites (hereafter “private land only”) and using BLM multiple use land as well as private land as options (hereafter “mixed ownership”). To ensure that the mitigation areas selected had relatively minimal degradation, we used an index of anthropogenic disturbance (road density, urban and agricultural land) adapted from Randall et al (2010) to define conservation suitability as the “cost” layer input for Marxan. The details of this layer and the input data are shown in Supporting Information S3. Using this cost layer in the Marxan mitigation scenarios provided a basis for comparison of the relative habitat quality available using the two sets of allowable land ownerships for mitigation.

For desert tortoise habitat distribution, we used the output of the habitat model developed by Nussear et al. (2009) and selected the top four scores (>0.6) of the classified output as a conservative representation of higher quality habitat [49]. For Mohave ground squirrel, we used the boundaries of the conservation areas as designated by the BLM in California [50].

**Table 4:** Compensation ratios for current and future mitigation scenarios.

Target	Current Ratio	Future Ratio
Species	3∶1	6∶1
Vegetation Systems	2∶1	4∶1
Unvegetated Systems	1∶1	2∶1

Mitigation ratios represent the proportional offset needed per unit of impact. Current Ratio refers to a hypothetical degree of offset to compensate for impacts to the target species or system based on a set of proposed projects. Future Ratio refers to a potential amount of mitigation that might be needed based on future build out of solar projects. Unvegetated systems include dunes, cliff and canyon, desert pavement, and playas.

## Supporting Information

Supporting Information S1 RETI Category 1 Exclusion Areas.
(PDF)

Supporting Information S2 Marxan settings and target amounts for compensatory mitigation scenarios.
(PDF)

Supporting Information S3 Process for determining disturbance value for cost layer used in mitigation scenarios.
(PDF)
