# Supplementary material for: An Approach to Enhance the Conservation-Compatibility of Solar Energy Development
Source: PLoS One. 2012 Jun 7;7(6):e38437. doi: 10.1371/journal.pone.0038437 (PMC3369905; doi:10.1371/journal.pone.0038437)
Supplement: Supporting Information S1 — RETI Category 1 Exclusion Areas. (PDF) [file pone.0038437.s001.pdf]

Supporting Information S1

RETI Category 1 Exclusion Areas

1. Designated federal Wilderness Areas and Wilderness Study Areas (WSAs)
2. California State Wilderness Area
3. Units of the National Park System
4. USFS Inventoried Roadless Area
5. National historic and scenic trail
6. National wild, scenic and recreational river- existing, proposed and study area
7. National Wildlife Refuges and USFWS Wildlife Management Areas
8. California state parks
9. California DFG wildlife areas and ecological reserve
10. BLM National Conservation Area
11. Private preserves of The Wildlands Conservancy
12. BLM national monuments
13. Existing conservation and mitigation banks under conservation easements approved by the DFG, FWS or Army Corps of Engineer
14. State owned land
