# Supplementary material for: An Approach to Enhance the Conservation-Compatibility of Solar Energy Development
Source: PLoS One. 2012 Jun 7;7(6):e38437. doi: 10.1371/journal.pone.0038437 (PMC3369905; doi:10.1371/journal.pone.0038437)
Supplement: Supporting Information S2 — Marxan settings and target amounts for compensatory mitigation scenarios. (PDF) [file pone.0038437.s002.pdf]

Supporting Information S2

Marxan settings and target amounts for compensatory mitigation scenarios

Software version: 1.8.10

Number of runs: 100

Number of iterations: 10,000,000

Boundary length modifier: .05

Temperature decreases: 10,000

Initial Temp: Adaptive

Cooling factor: Adaptive

Cost threshold not used

Table S2.1 Mitigation scenario target amounts for current and future scenarios. The species penalty factor is a parameter is adaptively set to ensure targets are met for all features. Other parameter values (type, target2, sepdistance, sepnum, targetocc) were set to 0. The goal amounts are known as "targets" in Marxan terminology, as distinct from targets as used to describe conservation features in this paper.

**Name**	**Subregion**	**Current goal (ha)**	**Future goal (ha)**	**Species penalty factor**
Annual Grassland	Western	202	404	1
Chaparral	Western	272	544	100
Cliff and Canyon	Western	57	114	1
Desert Pavement	Western	6	13	1
Dunes	Western	2	3	1
Mixed Salt Desert Scrub	Western	1,837	3,675	1
Mojave Mid-Elevation Mixed Desert Scrub	Western	5,286	10,572	1
Playa	Western	45	91	1
Southern Willow Scrub	Western	224	448	1
Mohave Desert Tortoise	Western	17,246	34,493	1
Mohave Ground Squirrel	Western	1,597	3,194	1
Annual Grassland	South central	6	13	1
Cliff and Canyon	South central	95	190	1
Desert Pavement	South central	3	6	1
Dunes	South central	198	397	1
Mixed Salt Desert Scrub	South central	628	1,255	100
Mojave Mid-Elevation Mixed Desert Scrub	South central	320	640	1
Playa	South central	29	57	1
Southern Willow Scrub	South central	140	280	1
Mohave Desert Tortoise	South central	15,388	30,776	1
Cliff and Canyon	Central	309	618	1
Desert Pavement	Central	119	238	100
Dunes	Central	137	274	1
Mixed Salt Desert Scrub	Central	321	642	1
Playa	Central	754	1,509	100
Southern Willow Scrub	Central	437	873	100
Mohave Desert Tortoise	Central	30,558	61,116	100
