# Supplementary material for: An Approach to Enhance the Conservation-Compatibility of Solar Energy Development
Source: PLoS One. 2012 Jun 7;7(6):e38437. doi: 10.1371/journal.pone.0038437 (PMC3369905; doi:10.1371/journal.pone.0038437)
Supplement: Supporting Information S3 — Process for determining disturbance value for cost layer used in mitigation scenarios. (PDF) [file pone.0038437.s003.pdf]

Supporting Information S3

Process for determining disturbance value for cost layer used in mitigation scenarios

The cost layer used in the Marxan mitigation site selection scenarios is a combination of a road disturbance layer where the weighted road density was calculated by weighting the class of roads (TIGER 2009) data and a land use disturbance layer of developed (urbanized) areas and intensive agriculture. The weights for the road density were assigned as shown in Table S3.

Assigned weights to TIGER road layer, using MFCC codes:

Table S3. Transportation categories and weights used in cost layer

Type	MTFCC	Weight
Railroad Feature (Main, Spur, or Yard)	R1011	3
Primary Road	S1100	9
Secondary Road	S1200	6
Local Neighborhood Road, Rural Road, City Street	S1400	3
Vehicular Trail (4WD)	S1500	1
Ramp	S1630	3
Service Drive usually along a limited access highway	S1640	3
Walkway/Pedestrian Trail	S1710	1
Alley	S1730	3
Private Road for service vehicles (logging, oil, fields, ranches, etc.	S1740	1
Private Driveway	S1750	1
Parking Lot Road	S1780	1
Bike Path or Trail	S1820	1

The LINEDENSITY function in ArcGIS was used to calculate the weighted road density with a kernel density type, 450 m search radius at a 90 meter cell size ([1]). This layer was normalized with values from 0-1 so that could be combined with the land use disturbance layer.

To calculate the land use disturbance levels we used two data sources, the NatureServe ecological systems data ([2]) and the California Department of Forestry and Fire Protection Multi-source land cover dataset ([3]). The NatureServe developed land and agricultural classes (21, 22, 80, 81, 82, 23, 24) were assigned a value of 1 and other values assigned a 0. This binary grid was combined with a layer of agricultural classes from the Calfire layer also at 90 m with agricultural types were also given a value of 1. The resulting grid was then smoothed using a FOCALMEAN function with a 9 cell (810 m) circular search radius.

The normalized road density and the smoothed land use layer were then added together with the land use layer being multiplied by 4 before they were summed. This was done to assign a higher weight to areas with converted land cover more than road density. To assign the values to the hexagon planning units for use in Marxan, we used the ArcGIS ZONALSTATS function to sample the cost layer with the planning units. We multiplied the mean cost value for each planning unit and multiplied it by 5000 to scale properly with the other Marxan cost factors.

1. ESRI (2009) ArcGIS v 9.3.1. Redlands, CA.
2. NatureServe (2009) National Ecological Systems dataset. Boulder, CO.
3. Calfire (2002) Multisource Land Cover dataset, fveg02.2. Calfire, Sacramento, CA.
